# Early-onset duodenal perforation during bevacizumab-containing chemotherapy in a patient with ovarian cancer: a case report

**DOI:** 10.1186/s40780-026-00608-9

**Published:** 2026-07-16

**Authors:** Satoshi Nagase, Keisuke Taguchi, Takanori Yoshida, Shokei Matsumoto, Yoko Toda, Hiroshi Kanno

**Affiliations:** 1 Department of Pharmacy, Saiseikai Yokohamashi Tobu Hospital, 3-6-1, Shimosueyoshi, Tsurumi-ku, Yokohama, Kanagawa 230-8765 Japan; 2 Department of Obstetrics and Gynecology, Saiseikai Yokohamashi Tobu Hospital, 3-6-1, Shimosueyoshi, Tsurumi-ku, Yokohama, Kanagawa 230-8765 Japan; 3 Department of Trauma and Emergency Surgery, Saiseikai Yokohamashi Tobu Hospital, 3-6-1, Shimosueyoshi, Tsurumi-ku, Yokohama, Kanagawa 230-8765 Japan; 4 Department of Palliative Medicine, Saiseikai Yokohamashi Tobu Hospital, 3-6-1, Shimosueyoshi, Tsurumi-ku, Yokohama, Kanagawa 230-8765 Japan

**Keywords:** Bevacizumab, Chemotherapy, Duodenal perforation, Anti-rheumatic therapy, Ovarian cancer

## Abstract

**Background:**

Bevacizumab-induced gastrointestinal perforation is a potentially fatal adverse event. However, duodenal perforations are rare. Conversely, patients with cancer often have concomitant rheumatoid arthritis and may be treated with a combination of anti-rheumatic drugs and symptomatic medications; however, the effects of these concomitant medications on bevacizumab-related perforation remain understudied. This report describes the case of a patient with ovarian cancer and a history of anti-rheumatic drug treatment who developed early-onset duodenal perforation during bevacizumab-containing chemotherapy, highlighting the importance of assessing the risk of duodenal perforation and the provision of multidisciplinary management.

**Case Presentation:**

The patient was a woman in her 50s with ovarian cancer that metastasized to the para-aortic lymph nodes. She was diagnosed with platinum-sensitive recurrence, and combination therapy with gemcitabine, carboplatin, and bevacizumab was initiated. She was also taking iguratimod and methotrexate for rheumatoid arthritis treatment. The patient developed sudden abdominal pain during the second course of chemotherapy and presented to the emergency department. A computed tomography (CT) scan revealed free gas, raising the suspicion of gastrointestinal perforation. Emergency laparotomy confirmed a duodenal perforation that was surgically covered with the ligamentum teres hepatis. A perforation of <1 cm was found in the anterior wall of the duodenal bulb, but the postoperative course was uneventful. The patient was discharged on postoperative day 7 and continued chemotherapy without bevacizumab. Postoperative upper endoscopy revealed an H2 stage ulcer.

**Conclusions:**

When administering bevacizumab to patients with ovarian cancer and multiple perforation risk factors, careful consideration should be given to evaluating existing ulcers, appropriately using concomitant medications, and modifying perforation risk factors to avoid serious adverse events. Therefore, collaboration between a multidisciplinary team, including oncologists, rheumatologists, and pharmacists, is crucial for appropriate drug use and continuous monitoring.

## Background

Gastrointestinal perforation caused by angiogenesis inhibitors is a serious adverse event that can occur in various areas of the stomach, small intestine, and large intestine [1, 2], although reports of such perforation in the upper gastrointestinal tract, such as the duodenum, are limited [3]. Particularly, gastrointestinal perforation caused by bevacizumab (BEV) has a mortality rate of 21.7% [4], making it a complication that should be avoided. The mechanisms underlying perforation development remain unclear, but factors such as intestinal mucosal ischemia, delayed wound healing, and tumor necrosis are considered possible mechanisms [5, 6]. Therefore, the prevention and management of gastrointestinal perforation require the identification of factors that cause mucosal damage and modification of perforation risk factors, early detection, and prompt intervention upon onset [7, 8].

Conversely, rheumatoid arthritis has been reported as a comorbidity among patients with malignant tumors or those undergoing chemotherapy [9], and these patients may be receiving concomitant medications. Among patients receiving BEV-containing chemotherapy, optimization of drug therapy is required, accounting for perforation risk factors, such as medications for comorbid conditions and pre-existing gastrointestinal lesions. However, the effects of each factor on BEV-related perforations in patients with multiple perforation risk factors remain understudied.

We report the case of a patient with ovarian cancer, comorbid rheumatoid arthritis, and a history of treatment with iguratimod (IGU) and methotrexate (MTX), who developed early-onset duodenal perforation during BEV-containing chemotherapy. This case is reported because it highlights the importance of assessing the duodenal perforation risk in patients receiving BEV, and the importance of multidisciplinary management.

## Case presentation

**Fig. 1:**
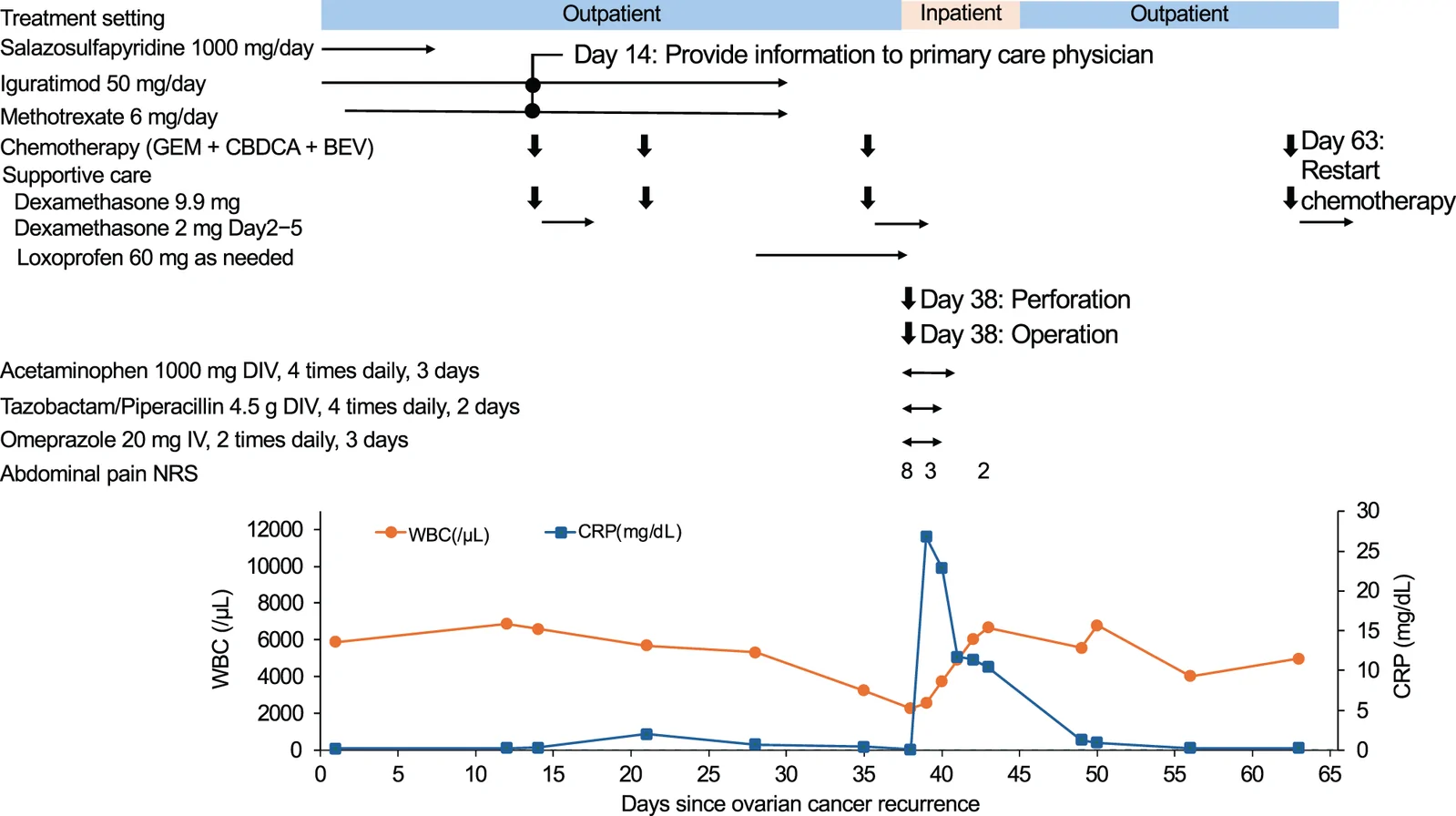
Summary of WBC, CRP, and clinical course of treatment. The x-axis indicates the number of days since ovarian recurrence, with illness day 1 representing the date of recurrent diagnosis. The y-axis shows WBC (/µL) and CRP (mg/dL). Solid circles (●) represent changes in WBC, and solid squares (■) represent changes in CRP. Chemotherapy consisted of gemcitabine, carboplatin, and bevacizumab administered on Day 1, followed by gemcitabine on Day 8; each cycle was repeated every 21 days. Supportive care included oral aprepitant (Days 1 − 3), palonosetron 0.75 mg administered intravenously on Day1, dexamethasone 9.9 mg and famotidine (20 mg) administered intravenously on Day 1, and oral dexamethasone at a dose of 2 mg/day on Days 2 − 5. Given the patient’s history of fatigue lasting for several days after anticancer drug administration during chemotherapy, oral dexamethasone was administered on Days 2 − 5. Loxoprofen was prescribed as needed, but was only administered a few times. GEM, gemcitabine; CBDCA, carboplatin; BEV, bevacizumab; NRS, numerical rating scale; WBC, white blood cell count; CRP, C-reactive protein

**Fig. 2:**
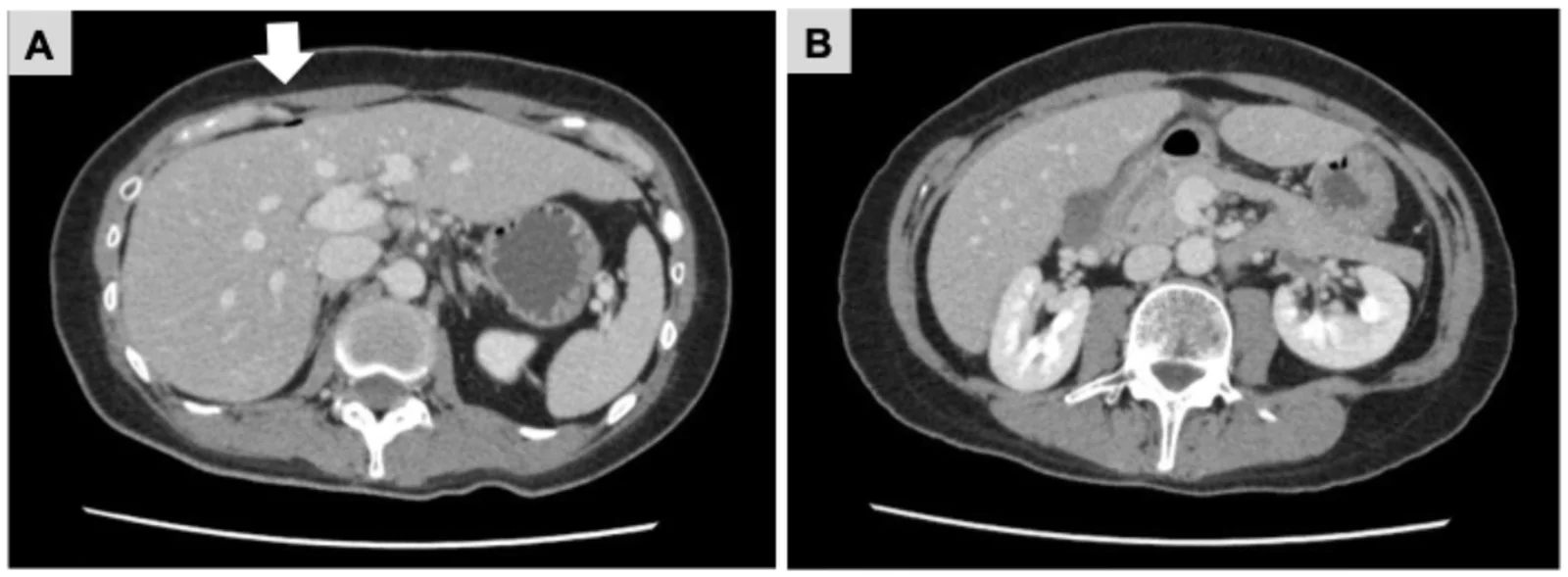
Computed tomography scan of a patient presented to the emergency room with abdominal pain. Image **A** reveals the presence of free air (arrow), and image **B** reveals thickening of the duodenal perforation bulb

**Fig. 3:**
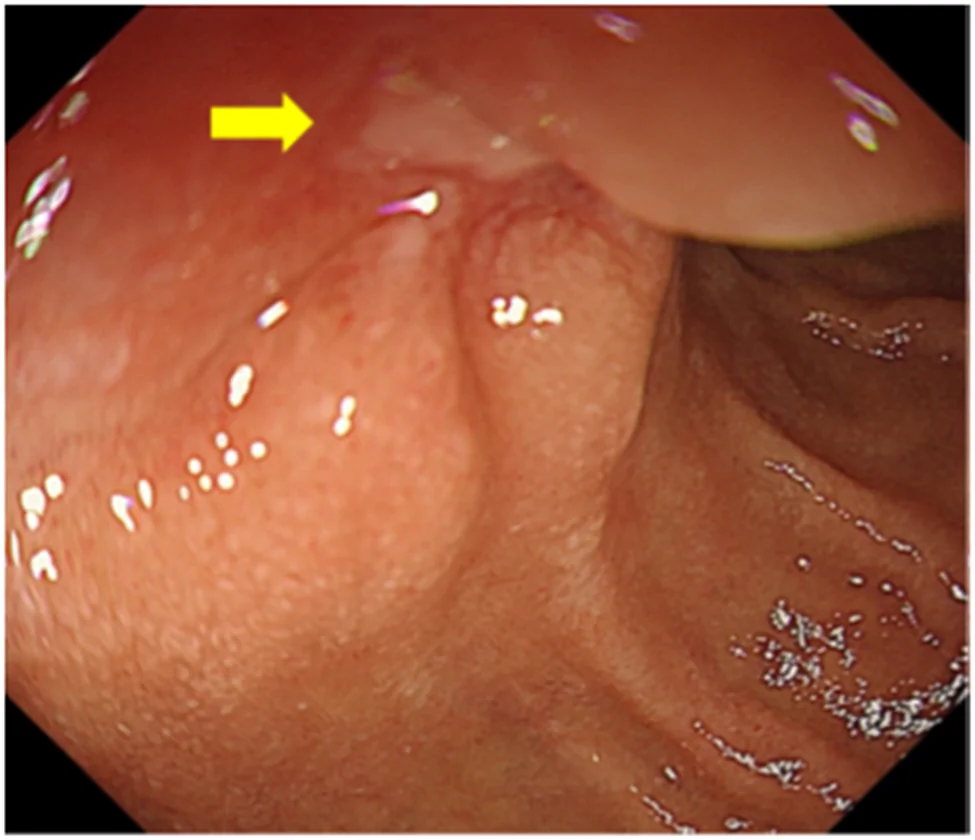
Endoscopic image showing an ulcer in the duodenal bulb. Postoperative endoscopic examination revealed an H2 stage duodenal ulcer (arrow indicates the ulcer area), and pathological results of the ulcer area showed no atypia or malignant findings

A woman in her 50s was diagnosed with left ovarian cancer and underwent bilateral malignant uterine adnexa tumor surgery and postoperative chemotherapy with paclitaxel (PTX) and carboplatin (CBDCA). Postoperative pathology revealed pT1c3N0M0 stage IC clear cell carcinoma. She had a history of metal (nickel) allergy, no history of smoking, peptic ulcers, diverticulitis, or colitis, and refrained from alcohol consumption. Hypersensitivity and anaphylaxis to PTX occurred during the first cycle of postoperative chemotherapy. Subsequently, hypersensitivity to docetaxel and liposomal doxorubicin hydrochloride developed; therefore, the regimen was switched to gemcitabine plus CBDCA (GEM + CBDCA), which was completed for seven cycles. One year and three months after completing postoperative chemotherapy, she was diagnosed with rheumatoid arthritis at another hospital. She continued taking the anti-rheumatic drugs IGU (5 mg twice daily) and MTX (6 mg once daily, once a week on Fridays) and maintained remission. One year and nine months after postoperative chemotherapy, para-aortic lymph node metastasis was detected on CT, and platinum-sensitive recurrence was diagnosed (day 1 of illness). The clinical course of ovarian cancer recurrence is shown in Fig. 1. On day 14 of the illness, the first cycle (21 days/cycle) of GEM (1000 mg/m^2^ on days 1 and 8) + CBDCA (area under the curve 5 on day 1) + BEV (15 mg/kg on day 1) therapy was initiated, targeting the recurrent lesions. On day 14 of illness, a medical information document was prepared by the physician and pharmacist regarding the details of chemotherapy, and the criteria for and feasibility of discontinuing anti-rheumatic drugs and the treatment policy of the attending rheumatologist was confirmed. On day 28, loxoprofen was prescribed to manage joint pain associated with chemotherapy. It was administered three times, with the last dose administered on the morning of the day when the perforation occurred. On day 30 of illness, IGU and MTX were discontinued as directed by the rheumatologist because the swelling and pain in the finger joints were under control. On day 38, the patient presented to the emergency department with sudden abdominal pain as the main complaint. A CT scan revealed free gas in the upper abdomen, raising the suspicion of duodenal perforation (Fig. 2). On the same day, emergency laparotomy was performed, which revealed a perforation of <1 cm in the anterior wall of the duodenal bulb, which was surgically covered with the ligamentum teres hepatis. On day 39 of illness, a decrease in the white blood cell count (2,270/µL) and an increase in the C-reactive protein (CRP; 26.8 mg/dL) were observed. The patient was admitted to the high care unit and transferred to the general ward the following day. Postoperative abdominal pain was rated as 8 on the Numerical Rating Scale (NRS), and acetaminophen 1,000 mg was administered four times daily for three days. Other drug therapies included tazobactam/piperacillin 4.5 g four times daily for two days and omeprazole 20 mg twice daily for three days. On day 45 of the illness, the patient was discharged on postoperative day 7 because of a reduction in abdominal pain (NRS 2), a decrease in CRP (10.4 mg/dL), and a favorable postoperative course at the surgical site. Pathological examination of the perforation site revealed no tumor infiltration, and postoperative upper endoscopy revealed an H2 stage ulcer in the duodenal bulb (Fig. 3). A pathological examination of the ulcer site revealed no signs of atypia or malignancy. Furthermore, the Helicobacter pylori antibody test results were negative. The patient had no history of accidental ingestion of press-through-package sheets containing medications, such as IGU, and no foreign bodies were detected during surgery or endoscopy. Owing to the development of perforation, on day 63 of the illness, the patient continued GEM + CBDCA therapy, excluding BEV, as the third cycle. After three cycles, slight shrinkage was observed in the abdominal aortic lymph nodes, and the rheumatoid arthritis remained in remission. Tumor growth was observed after 14 chemotherapy cycles.

## Discussion and conclusions

In the present case, duodenal perforation occurred early during BEV-containing chemotherapy for recurrent ovarian cancer. Furthermore, a distinctive feature of this case was the coexisting rheumatoid arthritis and concomitant use of conventional synthetic disease-modifying anti-rheumatic drugs (csDMARDs) at the start of the BEV treatment. No tumor infiltration or history of bowel resection was observed at the perforation site, and duodenal perforation was confirmed by postoperative endoscopy. However, the presence of a duodenal ulcer before BEV administration could not be confirmed, and it remains unclear whether the ulcer had been present before BEV administration or developed after BEV administration. The mechanism underlying the development of gastrointestinal perforation caused by BEV remains understudied [3, 10]. It is believed to involve vessel regression due to anti-vascular endothelial growth factor effects [10], ischemia of the intestinal mucosa [5], proliferation, and delayed healing of the intestinal wall [5, 10]. In the present case, these effects may have contributed to ulcer development [5, 11] or, if a pre-existing ulcer had been present, delayed healing of the ulcer, potentially increasing the risk of perforation. In the present case, the onset of perforation occurred 24 days after the first BEV administration, which is early-onset, as per a previously reported case of duodenal perforation in ovarian cancer at 10 days [3]. Furthermore, the median is within the ranges reported in previous studies, such as 71 days [7] and 3.35 months [12], suggesting that there is a temporal relationship. Furthermore, a Naranjo score of 5 indicated a “probable” causal relationship between BEV and duodenal perforation, providing supporting evidence for causality. These findings suggest that there is an association between BEV and duodenal perforation. However, reports of early-onset duodenal perforation during BEV administration are limited.

Previous studies have identified several risk factors for gastrointestinal perforation due to BEV, including intestinal obstruction and lesions such as chemotherapy-induced colitis, peptic ulcers, and drug-related factors [5, 6]. In the present case, because a duodenal ulcer was confirmed, the existing ulcerative lesion was highly likely to be a precursor to perforation. Furthermore, a previously reported unbalanced analysis demonstrated that anti-vascular endothelial growth factor drugs had a stronger gastrointestinal perforation signal than non-steroidal anti-inflammatory drugs (NSAIDs), indicating that their combined use is associated with a higher risk than either drug alone [13]. Although steroids have been reported to increase the likelihood of perforation by 1.6 times (odds ratio) [14], evidence for a dose-dependent perforation risk is limited. Additionally, because this patient did not develop gastrointestinal complications after resuming chemotherapy, it is unlikely that short-term dexamethasone use independently caused the gastrointestinal perforation. The patient was able to discontinue MTX and IGU eight days before the onset of perforation. The immunosuppressive effect of MTX may be enhanced when combined with chemotherapy, suggesting its potential impact on mucosal repair; however, its contribution to the onset of perforation is unclear. Gastrointestinal complications associated with IGU have been reported due to its cyclooxygenase-2 (COX-2) inhibitory effects [15, 16]. However, evidence demonstrating a direct association between IGU and duodenal ulcers and perforation is limited, making it difficult to assess. In the present case, salazosulfapyridine had been discontinued before the initiation of chemotherapy. In Japan, peptic ulcer disease and gastrointestinal perforation are listed as serious adverse reactions in the package insert for salazosulfapyridine. However, the published evidence supporting a direct causal relationship between salazosulfapyridine and these events, as well as their underlying mechanisms, remains limited. Therefore, although salazosulfapyridine may represent a potential background factor, its contribution to gastrointestinal perforation in the present case could not be clearly determined. Furthermore, a study reported that a BEV dose of 5 mg/kg/week carries a relatively higher and dose-dependent risk of perforation compared with 2.5 mg/kg/week [4]. In ovarian cancer, as in this case, a single dose of BEV (15 mg/kg) is equivalent to 5 mg/kg/week. Therefore, attention should be paid to the risk of gastrointestinal perforation. In the present case, BEV administration and the duodenal ulcer confirmed postoperatively, were strongly suggested to be associated with the development of perforation. On the other hand, multiple contributing factors, such as NSAIDs, steroids, MTX, and IGU were concurrently present, making it difficult to distinguish and evaluate the contribution of each factor. Although a direct causal relationship between the anti-rheumatic drugs and perforation could not be established in this case, the concomitant use of these agents may have represented one of the background factors to consider when assessing the risk of gastrointestinal perforation in the presence of other competing risk factors. Therefore, this case is considered to be an example that demonstrates the importance of comprehensively assessing the risk of gastrointestinal perforation in patients receiving BEV, and that these cases are managed through a multidisciplinary approach.

This patient was saved by rapid diagnosis and surgical treatment after perforation and achieved a favorable outcome similar to previously reported cases [17]. Chemotherapy, excluding BEV, was resumed from the third cycle and continued for approximately 10.3 months from the start of treatment until disease progression. The treatment duration in this case was similar to the reported progression-free survival (PFS) of 12.3 months for GEM + CBDCA + BEV [18]. Conversely, BEV contributes to an improved prognosis in ovarian cancer [19, 20], and discontinuation due to adverse events should be avoided as much as possible. Therefore, identifying and modifying risk factors is essential. This case offers valuable insights for avoiding serious adverse events. First, to appropriately determine whether to continue anti-rheumatic drugs and manage the adverse events caused by anticancer drugs, it is essential for oncologists and rheumatologists to share information and collaborate before initiating chemotherapy. Furthermore, as part of multidisciplinary collaboration, pharmacists play a crucial role in supporting individual physicians regarding the appropriate use of medications and the modification of perforation risk factors. Second, the importance of treating existing ulcers before BEV administration has been pointed out [8]. When using IGU or non-steroidal anti-inflammatory drugs (NSAIDs), it is important to administer prophylactic proton pump inhibitors or H2 blockers [5, 16] and to assess the presence or absence of peptic ulcers in advance. Third, in BEV-containing chemotherapy for ovarian cancer, acetaminophen should be considered instead of NSAIDs as a supportive therapy for anticancer drug-induced joint pain and fever. Although this finding is based on database studies, it suggests an association between NSAIDs and gastrointestinal perforation, regardless of COX-2 selectivity [13]. Considering the findings of this case, measures and close monitoring to prevent gastrointestinal perforation during BEV administration are required for patients with ovarian cancer and comorbid rheumatoid arthritis who are receiving BEV therapy.

This report has several limitations. In the present case, multiple perforation risk factors were present, including a duodenal ulcer, BEV, history of NSAIDs and steroid use, and history of MTX and IGU administration; therefore, it was difficult to distinguish and evaluate the contribution of each factor. In particular, there are limitations in estimating an independent causal relationship between perforation and anti-rheumatic drugs. Furthermore, the Naranjo score has limitations in evaluating adverse events involving multiple factors, and caution is required when interpreting the results.

In conclusion, this report describes the case of early-onset duodenal perforation during bevacizumab-containing chemotherapy in a patient with ovarian cancer, who presented with multiple risk factors for gastrointestinal perforation. Further accumulation of cases and investigations is required to clarify the association, and to determine the relative contribution of each risk factor to the development of perforation. To avoid serious adverse events, careful attention should be paid to the assessment of pre-existing ulcers, appropriate use of concomitant medications, and management of perforation risk factors. Therefore, multidisciplinary collaboration among oncologists, rheumatologists, pharmacists, and other healthcare professionals is important to ensure appropriate use of medications, and to provide continuous monitoring.

## Data Availability

No datasets were generated or analysed during the current study.

## Abbreviations

BEV: Bevacizumab
CBDCA: Carboplatin
COX-2: cyclooxygenase-2
CRP: C-reactive protein
csDMARDs: Conventional synthetic disease-modifying anti-rheumatic drugs
GEM: Gemcitabine
IGU: Iguratimod
MTX: Methotrexate
NRS: Numerical rating scale
NSAIDs: Non-steroidal anti-inflammatory drugs
PFS: Progression free survival
PPI: Proton pump inhibitor
PTX: Paclitaxel
WBC: White blood cell
